# A narrative review of ambulatory care education in Canadian internal medicine

**DOI:** 10.36834/cmej.69333

**Published:** 2020-12-07

**Authors:** Gillian Spiegle, Penny Yin, Sarah Wright, Stella Ng, Tara O’Brien, Farah Friesen, Michael Friesen, Rupal Shah

**Affiliations:** 1 Department of Medicine, University of Toronto, Ontario, Canada; 2 The Wilson Centre, University of Toronto, Ontario, Canada; 3 Centre for Faculty Development, Unity Health Toronto, Ontario, Canada; 4 Centre for Faculty Development, Faculty of Medicine, University of Toronto, Ontario, Canada

## Abstract

**Background:**

The Canadian healthcare system faces increasing patient volumes and complexity amidst funding constraints. Ambulatory care offers a potential solution to some of these challenges. Despite growing emphasis on the provision of ambulatory care, there has been a relative paucity of ambulatory care training curricula within Canadian internal medicine residency programs. We conducted a narrative review to understand the current state of knowledge on postgraduate ambulatory care education (ACE), in order to frame a research agenda for Canadian Internal Medicine ACE.

**Methods:**

We searched OVID Medline, Embase, and PsycINFO for articles that included the concepts of ambulatory care and medical or health professions education from 2005-2015. After sorting for inclusion/exclusion, we analyzed 30 articles, looking for dominant claims about ACE in Internal Medicine literature.

**Results:**

We found three claims. First, ACE is considered to be a necessary component of medical training because of its distinction from inpatient learning environments. Second, current models of ambulatory care clinics do not meet residency education needs. Third, ACE presents opportunities to develop non-medical expert roles.

**Conclusions:**

The findings of our narrative review highlight a need for additional research regarding ACE in Canada to inform optimal ambulatory internal medicine training structures and alignment of educational and societal needs.

## Abstract (fr)

**Contexte:**

Le système canadien des soins de santé fait face à des volumes croissants de patients et de cas complexes en même temps qu’à des contraintes budgétaires. Les soins ambulatoires offrent une solution pour relever certains de ces défis. Malgré l’importance grandissante portée aux soins ambulatoires, on observe un manque relatif de cursus de formation en soins ambulatoires dans les programmes de résidence en médecine interne. On a effectué une revue narrative en vue de comprendre l’état actuel des connaissances sur la formation en soins ambulatoires (FSA) postgraduée afin d’encadrer un programme de recherche portant sur la FSA à l’intention des étudiants en médecine interne canadiens.

**Méthodologie:**

On a consulté OVID Medline, Embase et PsycINFO pour trouver des articles publiés entre 2005 et 2015, portant sur les concepts de soins ambulatoires et d’éducation médicale ou des professionnels de la santé. Après la sélection d’articles selon des critères d’inclusion et d’exclusion, nous en avons examiné 30 en recherchant les affirmations dominantes sur la FSA dans la littérature en médecine interne.

**Résultats:**

On a dégagé trois affirmations soit 1) la FSA est tenue pour une composante nécessaire de tout programme d’études de médecine parce qu’elle se distingue de l’environnement d’apprentissage hospitalier; 2) les modèles actuels de cliniques de soins ambulatoires ne répondent pas aux besoins de formation des résidents; 3) la FSA permet de développer des rôles autres que ceux de l’expert médical.

**Conclusions:**

Les conclusions de notre analyse documentaire mettent en lumière la nécessité d’effectuer d’autres recherches sur la FSA au Canada pour connaître quelles seraient les structures optimales pour dispenser la formation en soins ambulatoires pour la médecine interne et établir une adéquation entre les besoins de formation et les besoins de la société.

## Introduction

While the patient population ages, caseloads increase, and complexity in care rises, healthcare systems also face pressures to decrease hospital admissions, shorten stays, and reduce readmissions.^1^ In response, healthcare systems at large are shifting to provide more care in the outpatient setting in an effort to reduce healthcare costs.^2^ These challenges have led to an increased commitment to ambulatory care, and necessary efforts to enhance ambulatory care education (ACE) within the field of internal medicine in both Canada and the United States.

Yet, despite increased emphasis on the provision of ambulatory care, there has been a relative paucity of ambulatory care training within Canadian internal medicine residency programs. Most internal medicine programs do not have a formal curriculum or relevant learning goals.^3^ Furthermore, General Internal Medicine (GIM) is a comparatively new subspecialty within internal medicine in Canada. The Royal College of Physicians and Surgeons of Canada (RCPSC) defined GIM as “a subspecialty of internal medicine which embraces the values of generalism, is aligned with population needs, and promotes the practitioner’s ability to adapt their practice profile when population needs change.”^4^^(p1)^ Given its nascent state, internists across Canada are attempting to define the role of ambulatory care within GIM, and how that can be reflected in its training.^5^^,^^6^

Most of the extant literature on this topic originates from the United States (US), but the field of internal medicine in Canada differs from the US in four main ways. First, the core internal medicine program is three years in the US, after which residents are eligible for independent practice.^7^ In Canada, three years of core internal medicine is followed by either an additional year in core internal medicine or 2-3 years of subspecialty training before residents are eligible to work as independent practitioners.^4^ Second, in Canada, GIM is considered a subspecialty of internal medicine, and requires an additional two years of training after the completion of core internal medicine.^4^ Third, internists who practice in an ambulatory setting in the US provide primary care, while the provision of primary care is predominantly reserved for family physicians in Canada.^4^^,^^7^ Instead, internists in Canada see patients on a referral basis from primary care physicians or other subspecialists.^4^ These patients often present with complex medical comorbidities.^8^^,^^9^ This results in internists in Canada seeing an overall different patient population mix than internists in the US. Fourth, the current structure of internal medicine in Canada allows for fewer opportunities for longitudinal care, where most internal medicine clinics are focused on rapid referrals from the emergency room or post-hospital discharge referrals for follow-up.^10^ These differences warrant a consideration of ACE specific to the Canadian context.

Despite existing distinctions in the practice of internal medicine between the US and Canada, both nations have seen a rise in ambulatory clinics to accommodate increasing patient volumes and chronic multi-morbidity. This clinical reality necessitates effective training of internal medicine residents. To support this goal, this narrative review aims to synthesize the current state of knowledge on postgraduate training in ambulatory internal medicine settings in order to frame a research agenda for ACE for internal medicine in Canada.

## Methods

To better understand the current state of ACE, we undertook a narrative review, which allows mapping of existing research and summarizing and consolidating the literature that exists.^11^ Adopting an iterative approach, our initial goals of the narrative review were to answer the following: *What is ACE responding to? For what and for whom is ACE happening? How and why did ACE develop?* As we progressed through each phase of data reduction, the research question and population of study became more refined: *What are the common claims in the literature on ambulatory care education in internal medicine?*

### Data sources and search strategy

We searched three databases (OVID Medline, Embase, and PsycINFO) for articles which included the concepts of ambulatory care and medical education or health professions education. The search was done on June 29, 2015 and used both subject headings and keywords. The subject headings for the concepts ambulatory care, education, medical education/health professions education were searched together (with “and”). The keywords captured the concepts for (ambulatory or teaching) and education. The search results were limited to 2005-2015 and English language only. A total of 2163 articles (Medline: 814, Embase: 1064, PsycINFO: 285) were exported to Endnote and deduplicated (338). The remaining 1825 articles were reviewed by title and abstract by two team members (SW, FF), with a third member (SN) who was consulted to resolve disagreements using pre-specified criteria.

### Eligibility criteria and screening process

At this stage of screening, articles were included if they were focused specifically on education in the ambulatory setting, resulting in 355 articles. Through ongoing discussion at group meetings, the research team continued to refine the focus and scope of the study and narrowed the inclusion criteria to articles specific to the medical profession (i.e. excluding articles focused on other healthcare professions, largely because in many other healthcare professions the bulk of patient care is delivered in ambulatory settings rendering the term less meaningful), articles that focused on postgraduate education and articles that helped us to better understand the purpose, usage and origins of ACE based on expert group consensus. This second screening resulted in the inclusion of 85 articles.

**Figure 1:**
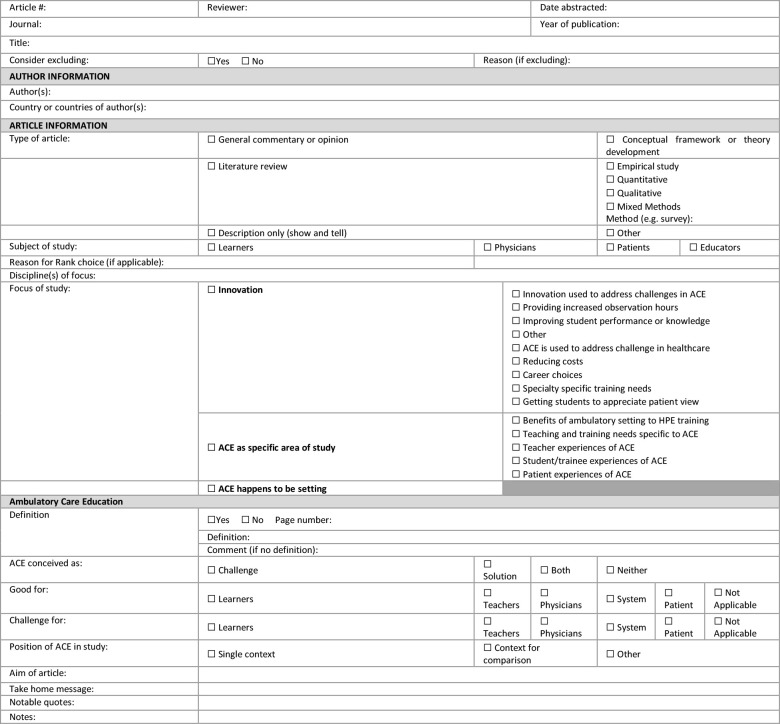
Abstraction sheet

Using an abstraction sheet created specifically for the study (Figure 1), full texts were reviewed and data collected from the 85 articles by two team members (SW and MF). Each full-text article was read and abstracted by at least two members of the team (SN, SW, MF). The abstraction sheet included article information (author, publication year, journal, author country/ies), article type, subject of study, focus of study, whether ACE was defined, conceptualization of ACE including claims (e.g. is ACE conceptualized as a challenge or benefit to learners, teachers, physicians, systems), discipline of focus (if applicable), any notable quotes, and additional notes. The abstraction process enabled us to take a closer look at the full texts and assess them for relevance to the research question. SW and MF agreed on the exclusion of 17 articles due to a lack of relevance to the study (for example, articles that included ACE but were focused on a specific training need within a specialty, or on a specific health policy only relevant within a particular political context, etc.). A further 25 articles were flagged for exclusion by only one reviewer. In such cases, a third reviewer (SN) made the final inclusion/exclusion decision, resulting in the exclusion of a further 21 articles due to lack of relevance.

**Figure 2:**
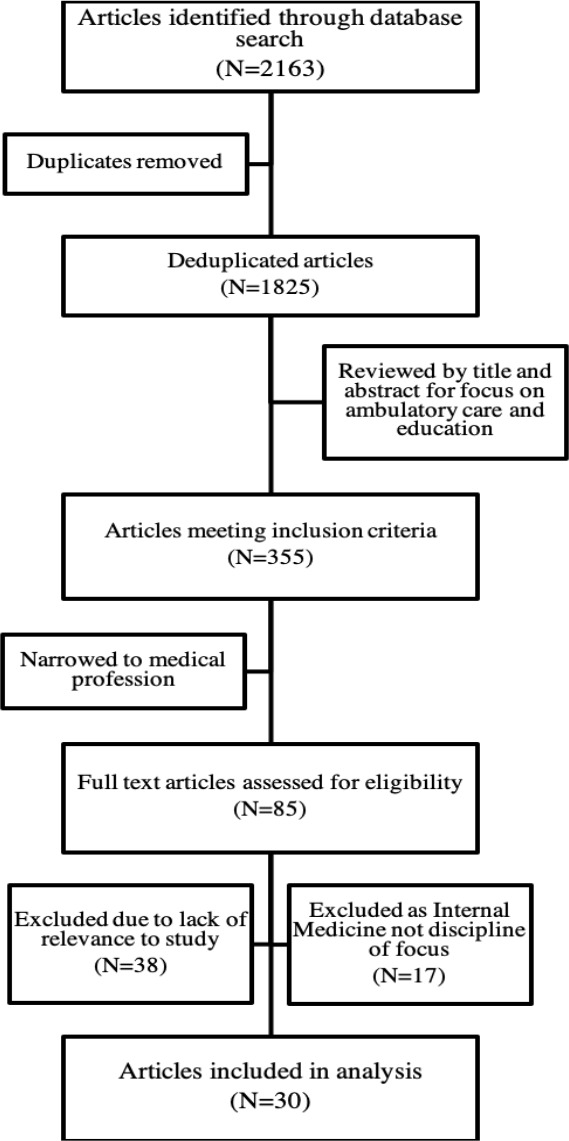
Study selection flow diagram

Following the full-text abstraction process, the team was able to take a holistic view of the dataset and further refined the focus of study on the claims made about ACE in internal medicine. Of the remaining 47 articles, 30 were focused on internal medicine mirroring the aforementioned shifts in the provision of care to ambulatory settings in response to growing patient volumes and multimorbidity.^1^ This gives credence to the notion that ACE has become a key educational priority for internal medicine, especially in Canada with the advent of GIM subspecialty training, warranting further study.^5^ We deliberately chose the 2005-2015 time frame to understand the literature in the five years preceding and following the recognition of GIM as a distinct subspecialty of internal medicine by the RCPSC in 2010.^5^ This decade spans a period where Canadian internal medicine residency programs were undergoing substantial organizational changes to launch 2-year subspecialty GIM programs. At the same time, the internal medicine patient population was changing in terms of higher volumes and complex multimorbidity that partly created the demand for ambulatory care within GIM.^1^ Our aim was to explore the literature to answer our research questions around ACE in the context of these rapid changes in GIM training and patient population characteristics. Seventeen articles were thus excluded as internal medicine was not a discipline of focus, leaving 30 articles for analysis. See Figure 2 for a flow diagram of article selection.

### Data summary and synthesis

Two members of the team (GS, PY) read each of the 30 full text articles to look for dominant or prevalent claims underlying scholarship about ambulatory care education in internal medicine. With specific attention to the claims about ACE made by the authors of the selected articles, GS and PY open coded each paper. Discussions of the coding structure took place during team meetings. Codes were discussed and findings synthesized, ultimately resulting in broad categories of the dominant and prevalent claims made in the literature. A summary of our findings identified through this iterative process are presented below.

## Results

We found 30 articles that met the inclusion criteria described above published between 2005 to 2015 (Table 1).^12^^-^^41^ The majority of the included articles (26 out of 30) were authored by US contributors.^12^^-^^24^^,^^26^^-^^29^^,^^31^^,^^32^^,^^35^^-^^41^ None of the articles were primarily authored by Canadians, with only one having contributions from Canadian educators.^20^ Only one of the articles defined ACE.^34^ The majority of articles focused on learners as their subjects of study. Most (28) of the articles specifically focused on internal medicine;^12^^-^^23^^,^^26^^-^^41^ whereas the other two included other specialties in addition to internal medicine.^24^^,^^25^ Twelve articles perceived ACE as a challenge,^16^^,^^21^^,^^23^^-^^25^^,^^27^^,^^29^^,^^30^^,^^32^^,^^34^^,^^40^^,^^41^ three as a solution,^17^^,^^22^^,^^28^ thirteen perceived ACE as both a challenge and solution^12-^^15^^,^^18^^-^^20^^,^^26^^,^^35^^-^^39^ and two described it neither in terms of a challenge nor solution.^31^^,^^33^

**Table 1:** Final articles for narrative review of ACE in Internal Medicine

Authors	Journal	Year	Title	Country	Discipline	Challenge/ Solution/ Both
Babbott SF et al	Acad Med	2010	Ambulatory office organization for internal medicine resident medical education	United States	Internal Medicine	Both
Bowen JL et al	J Gen Intern Med	2005	Changing habits of practice: Transforming internal medicine residency education in ambulatory settings	United States	Internal Medicine	Both
Chaudhry SI et al	J Gen Intern Med	2013	Moving forward in GME reform: a 4+1 model of resident ambulatory training	United States	Internal Medicine	Both
Colbert JA	N Engl J Med	2013	Experiments in continuity-rethinking residency training in ambulatory care	United States	Internal Medicine	Both
Cyran EM et al	J Gen Intern Med	2006	What do attending physicians contribute in a house officer-based ambulatory continuity clinic?	United States	Internal Medicine	Challenge
Fitzgibbons JP et al	Ann Intern Med	2006	Redesigning residency education in internal medicine	United States	Internal Medicine	Solution
Ganguli I	J Gen Intern Med	2014	Training for primary care's future	United States	Internal Medicine	Both
Gracey CF et al	Acad Med	2005	Precepting humanism: strategies for fostering the human dimensions of care in ambulatory settings	United States	Internal Medicine	Both
Holmboe ES et al	J Gen Intern Med	2005	Reforming internal medicine residency training: A report from the Society of General Internal Medicine’s Task Force for Residency Reform.	United States, Canada	Internal Medicine	Both
Huddle TS & Heudebert GR	Acad Med	2008	Internal medicine training in the 21st century	United States	Internal Medicine	Challenge
Julian K et al	Acad Med	2011	Creating the next generation of general internists: a call for medical education reform	United States	Internal Medicine	Solution
Kale SA & Barkin RL	Am J Ther	2008	Proposal for a medical master class system of medical education for common complex medical entities	United States	Internal Medicine	Challenge
Keirns CC & Bosk CL	Acad Med	2008	The Unintended Consequences of training residents in dysfunctional outpatient settings	United States	Internal Medicine/Paeds	Challenge
Lake FR & Vickery AW	Med J Aust	2006	Teaching on the run tips 14: teaching in ambulatory care	Australia	Not specified, but postgraduate	Challenge
Luciano G et al	Med Educ	2014	Training the ambulatory internist: rebalancing residency education	United States	Internal Medicine	Both
Lynn L et al	Health Affairs	2012	Gaps in quality of diabetes care in internal medicine residency clinics suggest the need for better ambulatory care training	United States	Internal Medicine	Challenge
Meyers FJ et al	Acad Med	2007	Redesigning residency training in internal medicine: the consensus report of the Alliance for Academic Internal Medicine Education Redesign Task Force	United States	Internal Medicine	Solution
Nadkarni M et al	J Gen Intern Med	2010	Ambulatory-based education in internal medicine: Current organization and implications for transformation. Results of a national survey of resident continuity clinic directors	United States	Internal Medicine	Challenge
Obara H et al	Med Educ	2007	The necessity for further reform in primary care training in Japan	Japan	Internal Medicine	Challenge
Papp KK & Wayne DB	J Gen Intern Med	2013	Ambulatory education redesign: time to get inspired	United States	Internal Medicine	Neither
Peccoralo LA et al	J Gen Intern Med	2013	Resident satisfaction with continuity clinic and career choice in general internal medicine	United States	Internal Medicine	Challenge
Junod Perron N et al	Educ Heal Chang Learn Pract	2009	Residents' perceived needs in communication skills training across in- and outpatient clinical settings	Switzerland	Internal Medicine	Neither
Junod Perron N et al	Swiss Med Weekly	2012	How to fulfill residents' training needs and public service missions in outpatient general internal medicine?	Switzerland	Internal Medicine	Challenge
Salerno S et al	Teach Learn Med	2007	Disruptions and satisfaction in internal medicine resident continuity clinic differ between inpatient and outpatient rotations	United States	Internal Medicine	Both
Sequist TD et al	Acad Med	2005	Use of an electronic medical record to profile the continuity clinic experiences of primary care residents	United States	Internal Medicine	Both
Sisson SD et al	J Gen Intern Med	2007	Continuity clinic satisfaction and valuation in residency training	United States	Internal Medicine	Both
Sisson SD & Dalal D	Am J Med	2011	Internal medicine residency training on topics in ambulatory care	United States	Internal Medicine	Both
Thomas KG et al	J Gen Intern Med	2009	Alternative approaches to ambulatory training	United States	Internal Medicine	Both
Warm EJ et al	J Gen Intern Med	2008	The ambulatory long-block	United States	Internal Medicine	Challenge
Zebrack JR et al	Am J Med	2010	Ambulatory training since duty hour regulations: a survey of program directors	United States	Internal Medicine	Challenge

In synthesizing the literature, we found three overarching claims.

### Claim 1: Ambulatory care education is necessary for medical education

The importance of ACE is reflected in a relatively recent increase in the mandatory requirements for ambulatory training from 16 to 33% in internal medicine residency training programs in the United States as per the Accreditation Council for Graduate Medical Education (ACGME).^24^ This is because the cohort of patients and disease presentations encountered in the outpatient setting are markedly different from its inpatient counterpart, demanding a diverse skill set and providing unique opportunities for learning and practice. Bowen et al. and Fitzgibbons et al. recognized these fundamental differences, particularly emphasizing the role of chronic disease management and longitudinal follow-up in ambulatory medicine.^13^^,^^17^ Authors shared the belief that postgraduate medical training should occur in settings that are concordant with one’s eventual practice setting.^25^^,^^27^ Specifically, authors recognized that the inpatient-focused training model no longer meets the current demands of the healthcare system in the face of expanding scopes and volume of ambulatory practice.^17^ Bowen et al. advocated that “experience is necessary for the development of diagnostic expertise and practice skills unique to ambulatory settings.”^13^^(p1182)^ To achieve this, arguments for residents to train in longitudinal clinics that look after patients with complex chronic medical comorbidities have been made.^13^^,^^15^

A need for exposure to chronic disease management was another reason for ACE. Lynn et al. advocated for the need to target residency training in clinics towards common high-impact and high-cost chronic diseases, as it would improve the overall quality of patient care received.^27^ As the demand for chronic disease management is on the rise, there have been respective efforts to focus training on skills that are required for future practice in ambulatory settings. Sisson & Dalal stated that “competent management of chronic diseases is a core skill of general internists, and as the prevalence of chronic diseases such as diabetes, hypertension, obesity, lipid disorders and others increase, more general internists will be needed.”^38^^(p89)^

### Claim 2: Current models of ambulatory care clinics do not meet residency education needs

The second overarching claim arising from the literature is that current models of ambulatory care clinics are insufficiently meeting residents’ training needs due to several factors such as disorganization, residents’ competing demands and clinic environments which do not consistently reflect authentic practice.

Firstly, organizational factors, such as the facilities, available personnel, clinic funding and competing faculty responsibilities may impede learning. Huddle & Heudebert reported that several ambulatory clinics do not have the required infrastructure, staff support and patient mix to provide a good ambulatory learning experience.^21^ Further, there are reports of residents becoming discouraged by administrative barriers that prevent them from providing the care that patients require.^24^ Specifically, Keirns & Bosk showed that administrative disorganization in primary care ambulatory clinics was likely related to decreased compensation for outpatient medicine, which in turn negatively impacted the training of future ambulatory care physicians.^24^ Ganguli reports that some academic sites were understaffed and poorly organized due to conflicting pressures for supervising physicians such as balancing research and clinical responsibilities.^18^

Competing demands between the inpatient and outpatient setting impede the ability of trainees to maximize learning in ambulatory clinics.^24^^,^^26^^,^^39^ Keirns & Bosk stated “When internal medicine residents are in their outpatient clinics, they are often distracted by calls requesting information, assistance, and orders for their very sick inpatients. The problems of the patient in the office with an upper respiratory infection take the resident away from a patient in the hospital with a new diagnosis of cancer. No matter how unfair the comparison, the problems of the outpatients are generally less compelling than the urgent needs of the inpatients”.^24^ ^(p500)^ Residents often felt obligated to attend to their responsibilities on the ward, making it difficult to focus on patient care in the ambulatory clinic, with the patients on the ward often “winning” the resident’s attention.^14^^,^^35^ In an attempt to solve this problem, one suggestion is that programs create an ambulatory block rotation, rather than having residents attend longitudinal clinics several half days per week while simultaneously working on inpatient wards, enabling trainees to immerse themselves in the provision of ambulatory care free from ward distractions.^21^^,^^32^^,^^40^ It is suggested that these blocks would need a well-defined syllabus with built in assessments to confirm the resident is competent in ambulatory care.^28^

Within current clinic models, residents are often described as dissatisfied with the experience as it does not mimic authentic ambulatory internal medicine practice. For example, Huddle & Heudebert reported that the “Continuity clinic does not recreate the satisfactions of internal medicine outpatient practice and attract trainees toward such practice. It immunizes them against outpatient internal medicine by providing a caricature of such practice, offering ‘continuity’ but providing a clinic experience that is, in fact, fragmented and frustrating both to trainees and to patients.”^21^^(p914)^ This sort of clinic atmosphere is a significant hindrance to the educational experience in an ambulatory clinic.^29^ In the US, disorganized clinic experiences leads to internal medicine residents feeling “frustrated” with ambulatory care instead of being motivated to learn in this environment, which is an issue provided that a significant number of patient encounters happen as an outpatient once residency training is completed.^37^ Adequate mentorship from internists with expertise in ambulatory medicine and exposure to highly functioning clinics are key to fostering resident interest in outpatient care.^22^^,^^23^

### Claim 3: Ambulatory care education presents opportunities to develop non-medical expert or intrinsic roles

The third predominant claim found in this narrative review is that ACE provides an opportunity for internal medicine residents to develop their non-medical expert, or intrinsic roles as physicians. These include health advocacy, manager, communication, and collaboration, amongst others as outlined in the CanMEDS outcomes-based framework.^42^

The ambulatory setting provides an opportunity to work with a broad range of patients, often in an interdisciplinary team, which allows the resident to hone in on the skills of collaboration, teamwork and use of health information technology.^27^ It is in this setting that residents are given more opportunities to work on these skills, participate in experiential learning and reflect on their experiences to further develop such skills.^33^ Outpatient clinics also enable residents to develop relationships with allied healthcare professionals, to learn how to manage clinic workflow and to receive feedback on how they communicate and function as a physician in the outpatient setting.^18^

The ambulatory setting provides a unique experience for residents to refine their skills in humanism and health advocacy. Humanism has become progressively imperative within medical education.^19^ As Gracey et al. states, “The ambulatory clinic as an environment for teaching presents unique opportunities as well as challenges for conveying humanism. The longitudinal relationships that develop between many preceptors and residents in outpatient settings are an important facilitator of teaching humanism.”^19^^(p26)^ Due to the longitudinal exposure to patients in many ambulatory clinics, residents simultaneously develop their interpersonal skills such as building productive patient rapport, handling the emotional context of patient visits, using counseling techniques to encourage behavioural change, and managing difficult patient situations.^24^

## Discussion

As healthcare systems across Canada and the US adapt to the changing landscape of an increasingly complex and volume-burdened patient population, greater provision of care in outpatient settings is needed. While the scope of ambulatory internal medicine practice may differ between the US and Canada, both nations face similar population health needs including increased multimorbidity.^8^^,^^9^ As a result, ACE should be a key priority for internal medicine residency training programs. Several of our initial questions remain unanswered, but the findings of our narrative review summarize three key claims made about ACE specific to the discipline of internal medicine. None of the studies reviewed were based in Canada, thus differences in scope of practice must be accounted for when applying findings across contexts. To be useful for generating a research agenda for Canadian internal medicine residency programs, these claims need to be thoughtfully considered and critically reviewed against a Canadian context. Gaps in the existing literature must also be accounted for.

The first claim identified is that ACE is necessary for medical education. While ambulatory care in the US tends to focus on primary care, ambulatory internists in Canada provide subspecialty care on a referral basis. Despite these differences in scope of practice, the first claim still resonates well within a Canadian internal medicine context. This is especially true since GIM has been recently recognized as an official subspecialty by the RCPSC since 2010, which provides a targeted focus on both ambulatory and acute care management.^5^ Additionally, the demand and nature of practice for the ambulatory internist is changing as patient volumes and complexity increase.^5^ Despite this societal need, the Royal College only mandates one ambulatory block within the first three years of core internal medicine residency training, which approximates to 2.6% of a resident’s entire duration of training,^4^ creating discordance between training needs in preparation for independent practice and accreditation standards. While a commitment to ACE is evident and necessary, further exploration of discordances in accreditation standards and training requirements is warranted.

A related gap in the current literature is that systematic efforts to define competencies relevant to ACE were not apparent for Canadian training programs. While some articles stressed that outpatient clinics should focus on longitudinal follow-up and chronic disease management,^13^^,^^17^ this may not apply equally to the Canadian context where primary care is provided predominantly by family physicians, not internists, and where a greater demand for rapid referral and post-discharge clinics over longitudinal clinics exists. Similar research efforts to systematically define the role of the ambulatory internist within a Canadian context are needed to identify competencies relevant to ACE. Such efforts will help inform the design and implementation of ambulatory clinics for subspecialty GIM training programs to ensure that these defined competencies are adequately achieved by residents via the process of careful curriculum mapping.

Existing literature on ACE within internal medicine is populated with studies identifying organizational and structural impediments to learning within outpatient training environments, leading to the second claim that current models of ambulatory care clinics do not meet residency education needs. While clinic funding models differ between the US and Canada’s universal healthcare system, organizational factors such as administrative barriers, competing faculty responsibilities and greater attention to inpatient responsibilities when paired simultaneously with ambulatory clinics,^18^^,^^21^^,^^24^^,^^35^ likely hold true in a Canadian context. Replication studies within subspecialty GIM ambulatory training environments in Canada would help confirm this claim. Fixing administrative barriers and adjusting demands on faculty who attend in clinic while busy with competing responsibilities, may be difficult to accomplish in ACE as these issues often stem from financial hindrances. Different incentive models (promotional or funding based) should be explored to encourage academic faculty to take on greater clinical and teaching roles in the outpatient setting.

Other barriers to ACE include competing demands placed on residents who rotate through interspersed clinics while caring for admitted patients. Exploring various strategies to deliver training experiences in outpatient settings, such as the ambulatory block rotation,^21^^,^^40^ would be a fruitful area of further research for both US and Canadian settings. Specifically, exploring both faculty and resident perceptions of a block versus longitudinal ambulatory rotation would help address if and how rotation structure impacts ACE. Comparisons of residents’ knowledge of common outpatient scenarios following block versus longitudinal rotations can be made, as the latter may reduce distractions imposed by simultaneous inpatient responsibilities.

In Canada, the use of rapid referral clinics to offload the number of consults seen in emergency departments, and the use of post-discharge clinics to decrease hospital length of stay, differ from the typical longitudinal clinics discussed in the articles covered in this narrative review. Patients seen in these Canadian clinics often present with issues of higher acuity compared to longitudinal clinics, and provide different educational contexts for trainees. However, these clinics are still likely to be plagued by the same organizational barriers identified in this review, which could be substantiated by replication studies in this context. Further research exploring residents’ perceptions of their educational experiences in a variety of clinics (i.e. rapid referral, post-discharge and longitudinal) may unveil how patient acuity and presentation alters perceptions of derived educational value. If clinics with higher patient acuity correlate with perceived educational value, for example, emphasis should be placed on helping residents link workplace-based experiences in lower acuity settings to real-life practice to improve perceptions of educational value in such settings.

The third claim that emerged from this narrative review is the notion that ACE presents opportunities to develop non-medical expert roles. Several differences between ambulatory and inpatient learning contexts exist that would support this claim. Given relative stability of patients in ambulatory environments, more time may be allotted to patient counselling and advocacy around conditions and/or medications, fostering communication skills.^19^^,^^24^ Longitudinal clinics may enable residents to forge long-term relationships with patients over time aligning well with the development of ‘intrinsic’ roles.^18^^-^^20^^,^^33^ Research to assess whether the same opportunities hold true in more ‘urgent’ clinics such as rapid referral and post-discharge, emerging in Canadian settings, would be important to assess the legitimacy of this claim across contexts and patient presentations.

On inpatient wards, trainees control their time and prioritize patients to be seen based on urgency and need. Managerial roles differ in the outpatient setting, where trainees must become adept at directing patient flow, clinic efficiency and follow-up as patients are booked to be seen at specific times.^43^ Studies to assess how these differences in the manager role, specifically trainees’ control over how to structure their day, may yield interesting insights into how patient flow impacts learning and education in both US and Canada.

## Limitations

There are several limitations with our study. First, the majority of the articles analyzed education frameworks and settings from an American context. Given differences in scope of practice between internal medicine in Canada and the US, some of the themes (such as primary care) are less applicable to the Canadian system. However, conducting the review enabled us to highlight the paucity of Canadian research in this area and outline a research agenda for the Canadian context. Second, the articles studied in this review are very heterogeneous in their sample size and target interventions, making it difficult for one to analyze and synthesize common trends. Third, ACE is a broad topic that does not have a specific definition in the literature. Even in our review, only one out of 30 articles provided a definition for what ACE means in the post-graduate setting.^34^

### Next steps

While this narrative review reveals prevalent claims that exist with respect to ACE in internal medicine, many questions remain, creating avenues for further research. Much of the literature available focuses on ACE in the US setting, which cannot be directly applied to other countries. Research to define the scope of practice of ambulatory care for Canadian internists is required to develop and refine educational competencies in this setting. Furthermore, workplace-based training should reflect authentic work environments. We need to explore the conflicts and confluence between mandated training requirements and societal needs, as healthcare systems expand ambulatory care. This may help program directors and educators develop curriculum to ensure that residents are adequately prepared for future career environments. Other opportunities for research in ACE include exploring and evaluating different rotation structures, such as dedicated block rotations, as well as differences between clinic type in outpatient settings. Lastly, ACE research to explore the development of non-medical expert skills and competencies, and how this can be harnessed to improve resident education in such settings, may increase interest for career development in ambulatory internal medicine.

## Conclusion

In this article, three prominent claims were discovered and discussed–ACE in internal medicine is necessary, the current model of ambulatory clinics are not meeting training needs of residents, and ACE in internal medicine allows residents to learn intrinsic, non-medical expert roles. This article highlights key claims that currently exist in the literature about ambulatory care education, and opportunities for further research in this area. While focused on internal medicine, many of these findings are widely applicable, serving as a springboard to foster discussion of education in ambulatory environments across disciplines and contexts.
